# The Effect of Task Demand on EEG Responses to Irrelevant Sound and Speech in Simulated Surgical Environments

**DOI:** 10.1111/psyp.70360

**Published:** 2026-07-14

**Authors:** Marc Rosenkranz, Verena N. Uslar, Dirk Weyhe, Martin G. Bleichner

**Affiliations:** ^1^ Neurophysiology of Everyday Life Group, Department of Psychology Carl von Ossietzky Universität Oldenburg Oldenburg Germany; ^2^ Pius‐Hospital Oldenburg, University Hospital for Visceral Surgery Carl von Ossietzky Universität Oldenburg Oldenburg Germany; ^3^ Research Center for Neurosensory Science Carl von Ossietzky Universität Oldenburg Oldenburg Germany

## Abstract

Complex soundscapes in high‐stakes environments, such as the operating room (OR), are characterized by a variety of overlapping auditory stimuli and present significant challenges for personnel, particularly during periods of high demand. This study investigates how task demand and an OR soundscape including irrelevant speech influence perceived workload, surgical performance, and auditory processing in a simulated surgical environment, using mobile electroencephalography (EEG). Participants performed two simulated surgical tasks, namely peg transfer and suturing, representing a low‐demand and high‐demand task, respectively. The tasks were performed under two sound conditions: an OR soundscape was presented with irrelevant speech or alone. Neural responses to transient and continuous auditory stimuli were analyzed using event‐related potentials (ERPs) and temporal response functions (TRFs), respectively. Results showed that irrelevant speech increased self‐reported workload, whereas task performance remained unaffected. EEG analyses revealed reduced neural tracking of the speech envelope under high task demand. This can be interpreted as a reduced processing of subjectively distracting sound that is not necessary for task completion and may point to a successful suppression of irrelevant speech. Notably, an inverse relationship was observed between neural responses to speech and self‐reported workload, indicating that the speech responses may serve as a marker for perceived workload. Overall, this study demonstrates the potential of EEG to assess irrelevant sound processing in realistic work‐like settings and highlights the critical role of task demand in modulating neural responses and self‐reported workload to soundscapes.

## Impact Statement

This study advances our understanding of auditory distraction in complex work environments by examining how operating room soundscapes, including irrelevant speech, interact with task demand. Using event‐related potentials (ERPs) and temporal response functions (TRFs) derived from mobile EEG, we show that high task demand is associated with reduced neural tracking of continuous speech. This finding highlights the role of task demand in shaping auditory processing and perceived workload in realistic work settings and demonstrate the potential of mobile EEG for studying auditory distraction outside the laboratory.

## 1 Introduction

Certain professions require a high degree of skill and precision while being performed in environments where mistakes can have severe consequences. Surgery, for example, is inherently demanding, requiring precise handling of instruments, advanced technological skills, and sustained concentration over long periods of time. In such professions, cognitive resources must be continuously allocated to incoming information across multiple sensory modalities (Wickens 2008).

The operating room (OR) is characterized by a complex auditory environment, comprising a multitude of concurrent sounds such as team communication, alarms, monitoring signals, and instrument use. Some of these sounds are directly relevant to the task at hand, whereas others are unrelated to the ongoing procedure. Processing and, when necessary, suppressing irrelevant auditory input within this complex soundscape places additional demands on cognitive control. As a result, irrelevant sounds in the OR are often experienced as distracting and have been linked to increased stress, reduced well‐being (Kern et al. 2019), and a higher likelihood of errors (Mentis et al. 2016). Thus, in this context, the term *distraction* can refer to two related but distinct phenomena. On the one hand, distraction may manifest as failures of attentional control that interfere with task execution and lead to measurable behavioral consequences, such as reduced efficiency or increased errors (objective distraction). On the other hand, distraction may reflect the subjective effort or stress associated with suppressing irrelevant information in order to maintain task performance (subjective distraction). Distinguishing between these two aspects is particularly important in complex, real‐world environments like the OR, where performance may be preserved at the cost of increased mental effort.

Although a variety of sounds can be perceived as distracting (Gülsen et al. 2021; Tsiou et al. 2008; Weigl et al. 2015), irrelevant speech, that is, speech unrelated to the procedure, is associated with higher subjective distraction (Healey et al. 2007; Tsiou et al. 2008; van Harten et al. 2021) and workload (Weigl et al. 2015; Wheelock et al. 2015). Although speech is frequently identified as a distractor in the OR, there is mixed evidence regarding the impact of the OR soundscape, including speech, on surgical performance—a measure of objective distraction. Some studies showed performance reductions when the soundscape was compared with silence (e.g., Pluyter et al. 2010; Siu et al. 2010) or when irrelevant speech is presented in isolation (Czerwiec et al. 2024). Yet, a realistic OR environment includes multiple overlapping sounds, with silence being a rare condition and irrelevant speech only one of several potential auditory distractors (Gülsen et al. 2021). This highlights the need to study how the combination of irrelevant speech and other overlapping sounds in the OR soundscape influence the individual and affect surgical performance under realistic conditions.

Instead of comparing an OR soundscape or speech with silence, auditory processing should be investigated in the context of varying task demands. Subjective reports from medical personnel indicate that irrelevant speech is perceived as particularly distracting during phases of high task demand compared to phases of low task demand (Persoon et al. 2011; van Harten et al. 2021). This suggests that surgical task demand modulates the impact of irrelevant speech distractions. Understanding the contribution of distractors like irrelevant speech and their interaction with task demands is therefore crucial for optimizing performance and improving the work environment.

A comprehensive assessment of the interaction between task demand and distraction in complex environments like the OR requires a combination of objective and subjective measures. Using only subjective measures (e.g., self‐reports), it can be challenging to separate the specific contribution of speech from the overall impact of the task demand, and other sounds in the OR environment (Dias et al. 2018). Similarly, measures of performance may not capture situations where individuals find the soundscape distracting, even if their performance is unaffected (Rosenkranz et al. 2024). For example, surgeons may experience increased workload in order to maintain a high level of performance within a distracting environment.

In addition to behavioral and self‐report measures, neurophysiological responses provide complementary information about how irrelevant auditory input is processed under varying task demands. To complement self‐reports and performance measures, electroencephalography (EEG) provides another measurement. Mobile EEG is increasingly employed in the study of work environments (Wascher et al. 2021) and provides reliable responses to complex soundscapes and speech while a task is being performed (Herrmann 2024; Rosenkranz et al. 2023, 2024; Xie et al. 2023; Korte et al. 2025). However, capturing EEG responses specifically related to the processing of irrelevant sounds requires a clear experimental manipulation; otherwise, it is difficult to determine whether a sound was truly distracting or merely processed. However, interpreting EEG responses to irrelevant sounds in complex, real‐world scenarios requires a clear experimental manipulation; otherwise, it remains unclear whether observed neural responses reflect general auditory processing, increased cognitive effort, or changes in attentional control. In this context, we related EEG to the different aspects of the soundscape like irrelevant speech or distinct sounds and assessed how the processing varied with task demand.

To investigate the neural processing of distinct auditory stimuli within the OR soundscape, we employed two complementary EEG analysis approaches. First, we computed event‐related potentials (ERPs) which are well‐established for studying the processing of transient sounds in relation to task demands (e.g., Wascher et al. 2021). This makes ERPs particularly well‐suited to examine how surgical task demand influences the processing of task‐irrelevant auditory stimuli. To account for the continuous aspects of the OR soundscape, we also computed temporal response functions (TRFs), a robust tool for capturing neural responses to continuous auditory stimuli, including speech and concurrent sounds (Crosse et al. 2016; Rosenkranz et al. 2023, 2024). TRFs capture the relationship between continuous auditory stimuli and neural responses using linear modeling. We applied a backward TRF approach, in which the stimulus envelope is reconstructed from the EEG signal by integrating information across channels and time lags. Reconstruction accuracy, defined as the correlation between the original and reconstructed stimulus envelopes, provides an index of neural tracking of the ongoing sound. By using both ERPs and TRFs, we aim to gain a more comprehensive understanding of how discrete and continuous auditory stimuli are processed and how task demand shapes the neural responses in a complex work‐like environment.

The ability to filter out irrelevant auditory information is essential for maintaining focus on a task. Sensory gating, a neural mechanism thought to help suppress responses to repetitive, irrelevant stimuli, may play a key role in this process (Lijffjt et al. 2009). To investigate sensory gating without interfering with the surgical task, we employed the paired‐click paradigm. This approach contrasts the neural response to an initial click to a repeated second click. The reduction in response from the first to the second click reflects the strength of sensory gating. Studies suggest that greater cognitive engagement in a task increases the response reduction in the paired‐click paradigm, indicating more effective inhibition of irrelevant stimuli (Lijffjt et al. 2009). Similarly, an increase in task demand can suppress the processing of irrelevant auditory stimuli when the relevant stimuli are of a different sensory modality (Molloy et al. 2019; Sörqvist et al. 2012, 2016). Sensory gating effects have been shown to remain robust even when individuals are engaged in cognitive tasks or exposed to background noise (Hölle and Bleichner 2023; Major et al. 2020), making this method interesting for studying auditory filtering under real‐world conditions. Based on these findings, we hypothesized that high task demand would enhance sensory gating, resulting in a larger difference between ERPs in responses to the first and second clicks compared to low task demand.

Using ERPs we investigated a specific aspect of sound processing, namely early responses to transient and irrelevant sounds (i.e., paired clicks). Although this is still a rather artificial stimulus, the natural soundscapes consist of distinct and concurrent, as well as, overlapping auditory events. To better capture how such soundscapes are processed, we computed a general neural response using TRFs. This approach allowed us to separately assess neural tracking of the concurrently and continuously presented OR sounds and speech.

We expected the prediction accuracy of the OR sounds and speech to be modulated by task demand, though with different expectations regarding the direction of effects. Most TRF studies showed that attention is a driving factor of prediction accuracies, with attended streams resulting in larger prediction accuracies than unattended streams (e.g., Ding and Simon 2012; Crosse et al. 2016; Hausfeld et al. 2018; Xie et al. 2023). Thus, a difficult task may engage more attentional resources than an easy task with fewer resources remaining to process sounds and speech, resulting in lower prediction accuracies for the difficult compared to easy task. However, speech has a high potential to distract (Szalma and Hancock 2011) and is particularly disruptive for OR personnel during phases of high task demand (van Harten et al. 2021; Weigl et al. 2015; Widmer et al. 2018). Since distracting stimuli can engage attentional resources (Huang and Elhilali 2020; Holtze et al. 2021), we considered the possibility that especially distracting speech during high‐demand phases would also result in larger prediction accuracies. Thus, while we expected lower prediction accuracies in response to the OR sounds for the difficult compared to easy task, we were less certain regarding the direction of effect for speech. To further explore how perceived task demand and sound processing relate, we examined the relationship between subjectively reported workload and prediction accuracy in response to continuous OR sounds and speech.

In summary, this study examined how an OR soundscape including irrelevant speech and varying task demands interact to influence self‐reports, surgical task performance, and auditory processing, as measured by EEG. We hypothesized that higher task demand will lead to an increase in perceived workload. Additionally, we explored the interaction between task demand and speech presence to assess their combined influence on self‐reported workload. As workload is a rather general measure of surgical task demand, we also explored how the tasks and speech affect specific aspects of self‐reported demand (Wilson et al. 2011). We further explored the effect of irrelevant speech on surgical task performance. Regarding neural responses, we applied two analytic approaches. We investigated early sound responses using ERPs and expected that higher task demand will result in an increased sensory gating, as measured by ERPs. We also investigated responses to the ongoing OR sounds and speech. We hypothesized that neural tracking of the OR playback would vary as a function of task demand, with lower reconstruction accuracy during a high‐demand compared to low‐demand task. Furthermore, we explored the relationship between task demand and speech processing, as well as the association between the neural tracking of the OR playback and speech with self‐reported workload.

## 2 Method

This study involving human participants was reviewed and approved by Medizinische Ethikkommission, Carl von Ossietzky Universität Oldenburg, Oldenburg (2021‐031). The participants provided their written informed consent prior to participating in this study. All participants received monetary reimbursement.

### 2.1 Participants

Twenty‐five participants were recruited through an online announcement on the University board (age range: 19–34; mean age = 24.84; 14 women; 10 medical students). Eligibility criteria included: self‐reported normal hearing, normal or corrected vision, no psychological or neurological condition, right‐handedness, and no experience with surgery or surgical simulations. In total, five participants were excluded from all analyses involving EEG due to the following reasons: no data were present due to a recording error (*N* = 1); package loss during recording resulted in timing problems (*N* = 2); connector problems resulted in artifactual data (*N* = 2). Those participants were still included in the self‐report and performance analyses.

### 2.2 Paradigm

**FIGURE 1:**
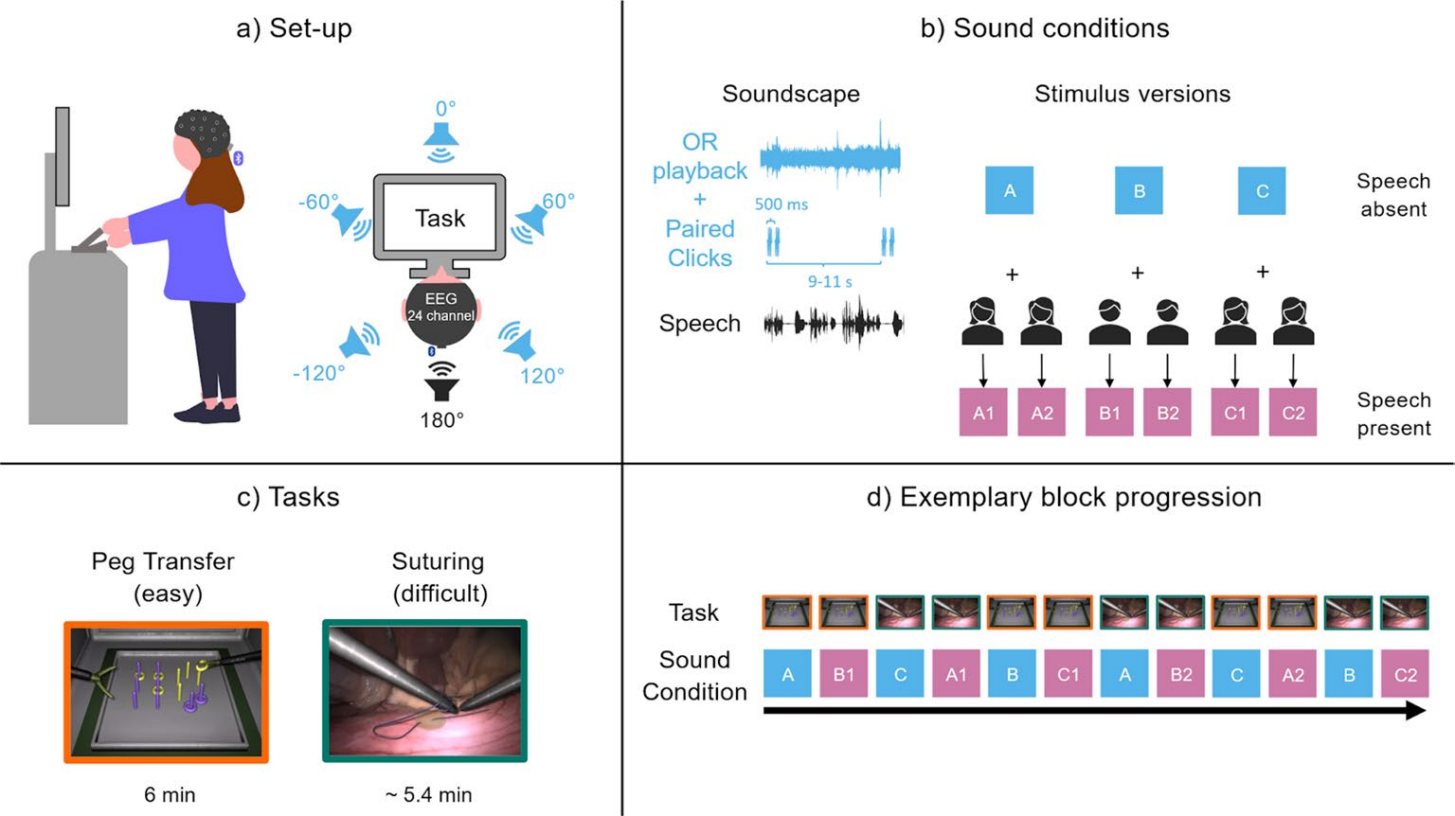
(a) Participants performed surgical training tasks while standing in front of a surgical simulator, equipped with a 24‐channel mobile EEG cap. A soundscape was presented through a loudspeaker array positioned around them. From five loudspeakers, marked in blue, an OR playback and paired clicks were presented. The five loudspeakers presented the same audio. From one loudspeaker, marked in black, speech was presented. (b) All sound conditions (i.e., speech‐absent and speech‐present) included the OR playback and paired clicks. Additionally, stories from three speakers (two stories per speaker) were presented, with each speaker paired with one of the three OR playbacks. This resulted in six stimuli for the speech‐present condition. (c) Participants completed two tasks of varying difficulty: Peg transfer (easy/low‐demand) and suturing (difficult/high‐demand). The peg transfer task was performed until the end of a block (after 6 min). The suturing task was performed until it was finished (which was on average after 5.4 min), but no longer than 6 min. (d) Example block progression for one participant. Each task was presented in two consecutive blocks, alternating between speech‐present and speech‐absent conditions. The starting task and sound condition were counterbalanced across participants. Additionally, while the overall order of sound conditions was fixed, the starting sound condition was rotated between participants.

We employed a within‐subject design, featuring a 2 (task: easy vs. difficult) × 2 (sound: speech present vs. speech absent) factorial structure, where each condition was repeated over three blocks, resulting in a total of 12 blocks (Figure 1). The participants were required to complete the surgical tasks peg transfer and suturing. These tasks were selected as they have been demonstrated to elicit either low or high workload, respectively (Lim et al. 2023; Scerbo et al. 2017). During all blocks, participants were presented with a task‐irrelevant soundscape. In all blocks the soundscape consisted of sounds from an actual OR (i.e., OR playback), and click sounds. Additionally, in half of all blocks, speech was presented. Each block lasted up to 6 min, with the end of the block signaled by the soundscape fading out, indicating that participants should stop the surgical task. Participants were instructed that all auditory stimuli were irrelevant and could be ignored.

#### 2.2.1 Auditory Stimuli

We generated nine auditory stimulus versions, each lasting 6 min, which were consistent across all participants (Figure 1b). Of these, three versions featured each a different OR playback with click pairs distributed throughout the playback duration and were used for the speech‐absent conditions. The remaining six versions were used for the speech‐present conditions, created by pairing each speech‐absent version with one of three speakers, with each speaker narrating two different stories. The OR playback and paired clicks were presented through five loudspeakers positioned around the participant (0°, 60°, 120°, −120°, −60°). Although the five loudspeakers presented the identical audio, speech was presented through a single loudspeaker positioned behind the participant (180°, Figure 1a). The distance between each loudspeaker and the participant was 2–2.5 m. Auditory stimuli were presented using Psychtoolbox 3 (v3.0.17; Kleiner et al. 2007). For each stimulus type, a sound marker was generated using the lab streaming layer library (v1.14; Kothe et al. 2024).

##### 2.2.1.1 OR Playback

The three OR playbacks were extracted from a recording during a visceral surgery, which was recorded using one field recorder which was positioned close to the surgery table at the University Hospital Oldenburg Rennies et al. (2023). The recording contains a variety of sounds, such as ventilation noise, beeps from monitoring devices, instrument clatter, and instrument sounds. Intelligible speech was removed after the recording for privacy reasons; however, unintelligible muttering and non‐vocal sounds such as coughing were preserved. The sound intensity of the OR playback was on average 55 dB(A).

##### 2.2.1.2 Paired Clicks

We presented pairs of clicks, with an interval of 500 ms between clicks and an interval of 9–11 s between click pairs. Each click pair consisted of two identical clicks (1000 Hz, 4 ms duration, 1 ms onset and offset ramps). In total, 35 pairs were presented per block. To ensure that energetic masking influences of the clicks were similar across participants, all pairs were presented at fixed moments in the OR playback.

##### 2.2.1.3 Speech Stimuli

The speech stimuli were chosen from a database containing German speakers, who were telling stories about self‐selected content (Daeglau et al. 2023), and have been shown to provide measurable EEG responses (Daeglau et al. 2025; Wiedenmann et al. 2023). The natural speech included speech pauses and filler words which increased the ecological validity of our approach. Three speakers were chosen, each telling two stories. To control for differences in loudness, the speech stimuli were matched to have the same root‐mean‐square (RMS) value. When speech was presented, the average sound intensity increased by 2–3 dB(A).

#### 2.2.2 Surgical Tasks

For the surgical task, the LabSim (Surgical Science, Sweden) simulator was used. The simulator includes the surgical training tasks peg transfer and suturing which were chosen as they differ in difficulty (Figure 1c; Lim et al. 2023; Scerbo et al. 2017), required bi‐manual control, and lasted at least 3 min for inexperienced individuals, ensuring that sufficient data could be collected. This minimum duration was an approximation based on observations from pilot data. As the tasks varied in their goal and procedural steps, the performance measures were different between tasks. Peg transfer included the performance measures “number of transfers” and “number of drops”, whereas suturing included the performance measures “duration” and “damage”. Although participants were not provided with any feedback regarding their performance during the task, they were instructed as to the performance measures that we investigated. Participants always completed two consecutive blocks of one task (e.g., peg transfer) and then switched to two consecutive blocks of the other task (e.g., suturing).

##### 2.2.2.1 Peg Transfer

In the peg transfer task, participants were required to transfer rings between two pairs of pegs, including a switch between grasping instruments for each transfer. This task was defined as easy, as it involved only a few and repetitive procedural steps. Participants were instructed to complete as many transfers as possible within a block with minimal ring drops. The task automatically ended after 6 min. The number of transfers and drops was used as the performance measures; these values were not directly provided by the LabSim software but could be computed based on the number of grasps, average drops, and average transfers.

##### 2.2.2.2 Suturing

In the suturing task, participants were required to drive a needle through tissue and tie two knots in the suture thread using the provided instruments. This task was more difficult than the peg transfer, as it included several procedural steps and required a higher degree of dexterity. Once the second knot was tied, the task ended and the soundscape stopped. The performance measures were task duration and damage. Damage was defined as the number of times the tissue was touched and the amount of pressure applied to the needle after it was driven through the tissue. This resulted in a damage score from 0 to 100, with a higher score indicating less damage.

#### 2.2.3 Counterbalancing of Blocks

To counterbalance the order of tasks, half of the participants started with peg transfer, whereas the other half started with suturing. The experiment followed a fixed order of stimulus conditions where speech absence and presence alternated with each block (Figure 1d). To counterbalance the sequence of stimulus conditions across participants, participants began at a different starting point within this fixed order and then continued sequentially. Consequently, the order was repeated after the 12th and 24th participant. We further ensured that one story of each speaker was presented during peg transfer and the other story of each speaker during suturing.

#### 2.2.4 Procedure

After arrival, participants practiced the use of the lab simulator for 45 min with the following procedure. To get acquainted with the simulator and the instruments, two simple training tasks (i.e., instrument navigation and grasping) were repeated twice each. This was followed by two blocks of peg transfer and two blocks of suturing. During the first block of each task, the experimenter provided instructions and guidance. During the second block of each task, the experimenter left the room and an OR playback was presented. The OR playback was not used during the experimental blocks. Participants were always allowed to ask questions and watch short instruction videos, provided by the manufacturer of the simulator. After the training, the EEG cap was fitted and participants performed resting measurements (i.e., 2 min of eyes open, 2 min of eyes closed, and listening to a sequence of 20 beeps). After these, participants performed the 12 experimental blocks. After each block participants completed the SURG‐TLX (Wilson et al. 2011) thereby providing a self‐reported workload measurement. The SURG‐TLX contains six items related to the different aspects of surgical demand (mental demand, physical demand, temporal demand, complexity of procedure, stress, and distraction). The items were rated on a visual analogue scale with scores ranging from 0 (low) to 20 (high). At the end of the experiments, participants answered 12 questions regarding the content of the speech stimuli (e.g., Was the topic of one of the stories a bike?). Six questions were related to the content of the stimuli—one question per stimulus—while six questions were unrelated. The participants had to indicate whether they perceived the content or not. An inspection of the speech content questions showed that 86.67% of the questions were answered correctly. Thus, participants could discriminate between speech‐related and speech‐unrelated questions.

### 2.3 EEG Data Acquisition

Participants were asked to wash their hair on the day of recording prior to the experiment. EEG data were recorded using a wireless amplifier (SMARTING, mBrainTrain, Belgrade, Serbia) attached to the back of a 24‐channel EEG cap (EasyCap GmbH, Herrsching, Germany) with Ag/AgCl passive electrodes and the reference and ground electrode at positions FCz and AFz, respectively. The data were recorded using a sampling rate of 500 Hz and transmitted via Bluetooth from the amplifier to a Bluetooth dongle (BlueSoleil) that was plugged into a computer (DELL Precision 3630). After fitting the cap, the skin at each electrode site was cleaned using 70% alcohol. Skin conductance between the scalp and electrodes was increased using abrasive gel (AbralytHiCl, Easycap GmbH, Germany). Impedance was kept below 10 kΩ at the beginning of the recording. The transmitted EEG data and sound marker were recorded in the Lab Recorder software (https://github.com/labstreaminglayer/App‐LabRecorder, v1.14) and saved as .xdf files. The same computer was used for data recording and sound presentation. Due to technical reasons, a constant delay between the marker indicating sound onset and the actual sound presentation was measured in advance. To account for this delay, the marker was adjusted offline by shifting it 40 ms.

### 2.4 EEG Preprocessing

The EEG data were analyzed using EEGLAB (v2022.1; Delorme and Makeig 2004) in MATLAB R2020b (The MathWorks, Natick, MA, United States). As a first step, bad channels that were recognized during recording were removed, resulting in the removal of one channel for two participants. After bad channel removal, the data were cleaned from artifacts using infomax independent component analysis (ICA). To improve ICA, the EEG data were filtered in two consecutive steps, first high‐pass filtered at 1 Hz and then low‐pass filtered at 40 Hz using zero‐phase Hamming‐windowed FIR filters (*pop_eegfiltnew*). The high‐pass filter (1 Hz) had 1651 taps, a transition bandwidth of 1 Hz, a passband edge at 1 Hz, and a −6 dB cutoff at 0.5 Hz. The low‐pass filter (40 Hz) had an order of 167 taps, a transition bandwidth of approximately 9.9 Hz, a passband edge at 40 Hz, and a −6 dB cutoff at 44.97 Hz. The filtered data were subsequently resampled to 250 Hz and used to compute the ICA decomposition. Then, the resting data and data of each block during which audio was presented were cut into consecutive epochs of 1 s. To minimize artifacts from the start and end of the task, the first and last 5 s of each block were excluded. Improbable epochs with a global (all channels) or local (single channel) threshold exceeding 5 standard deviations were automatically rejected using the *jointprob* function. ICA decomposition was applied to the remaining epochs. The resulting components were back‐projected on the raw data.

We continued with two filtering approaches depending on the ERP and TRF analysis. For ERP analysis, the raw data were first high‐pass filtered at 0.1 Hz and then low‐pass filtered at 40 Hz. The high‐pass filter (0.1 Hz) had an order of 16,501 taps, a transition bandwidth of 0.1 Hz, and a −6 dB cutoff at 0.05 Hz. The low‐pass filter (40 Hz) had an order of 167 taps, a transition bandwidth of approximately 9.9 Hz, a passband edge at 40 Hz, and a −6 dB cutoff at 44.97 Hz.

For the TRF analyses, EEG were first high‐pass filtered at 1 and low‐pass filtered at 30 Hz. The high‐pass filter (1 Hz) had 1651 taps, a transition bandwidth of 1 Hz, a passband edge at 1 Hz, and a −6 dB cutoff at 0.5 Hz. The low‐pass filter (30 Hz) had 221 taps, a transition bandwidth of 7.5 Hz, a passband edge of 30 Hz, and a −6 dB cutoff at 33.75 Hz.

The back‐projected components were then classified using the EEGLAB toolbox ICLabel (Pion‐Tonachini et al. 2019) with the ‘lite’ classifier which is better at detecting muscle artifacts than the default classifier (Klug and Gramann 2021). Components belonging to the categories eye blink and movement or muscle movement with 70% confidence or heart with 80% confidence were removed. Note, that the *ICLabel* classifier did not classify all components correctly because it was trained on stationary data with a larger electrode setup than ours. Therefore, we manually checked the components and made the following adjustments: Components indicating lateral eye‐movement were not always correctly classified and individually removed. Furthermore, channel Tp9 and Tp10 contained noise from muscle movement. We observed this already in a previous experiment, where a surgical simulator was used (Rosenkranz et al. 2024). We assume that the bi‐manual control of the simulator activates neck muscles, resulting in artifacts in electrodes that are close to the neck. As channel Tp9 and Tp10 are used for re‐referencing, we removed components showing muscle activity in Tp9 and Tp10. Note, that for the ERP and TRF analysis the same components were removed. Afterwards, previously rejected channels were interpolated using spherical interpolation. Lastly, channels were re‐referenced to the linked mastoids (Tp9 and Tp10).

### 2.5 ERP Analysis

**FIGURE 2:**
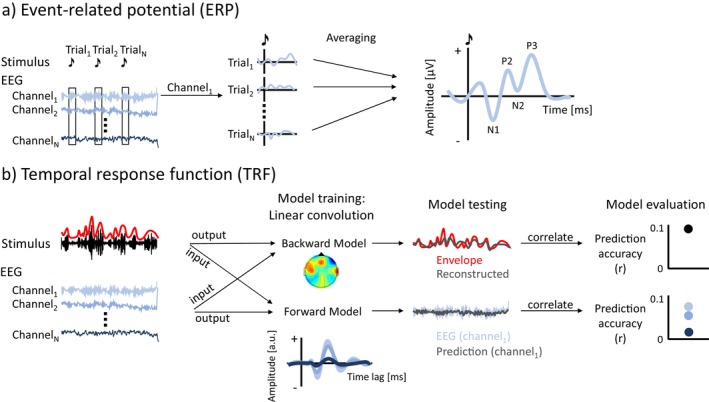
(a) In the ERP approach, neural responses are time‐locked to discrete stimulus onsets and averaged across repeated trials to enhance stimulus‐related activity and suppress unrelated EEG fluctuations. This averaging yields characteristic deflections (e.g., N1, P2, N2, P3) in the voltage time course. (b) In contrast, the TRF framework models the continuous relationship between a stimulus feature (e.g., the speech envelope) and the EEG using linear convolution. Forward models predict EEG responses from the stimulus, whereas backward models (as used in this manuscript) reconstruct stimulus features from multichannel EEG. Model performance is evaluated by correlating predicted and observed signals, yielding a measure of prediction accuracy. This approach enables the analysis of neural tracking in continuous, naturalistic stimulation without explicit trial segmentation.

We analyzed the neural response to the irrelevant soundscape using two different approaches. Event‐related potentials (ERPs) were computed in response to the paired clicks, whereas a temporal response function (TRF) was used to analyze the neural response to the OR playback and speech (see Figure 2 for a visual summary of the two methods). We quantified ERP amplitudes using the following procedure: For each block we extracted epochs from −200 to 1000 ms relative to the onset of the first click and baseline‐corrected the epochs from −200 to 0 ms. Epochs exceeding a threshold of three standard deviations globally (across all channels) or locally (within a single channel) were automatically rejected using the *jointprob* function. Data from channel FC1, FC2, Fz, and Cz were then averaged, as the auditory N1 and P2 ERP components are prominent at these channels (Crowley and Colrain 2004; Näätänen and Picton 1987). To extract the N1 and P2 components for both the first and second clicks, we first computed an average ERP across participants for each sound condition. It should be noted that the extraction of time windows was conducted for the two sound conditions separately to account for the acoustic differences between the two conditions. For each sound condition, we identified the N1 peak within the range of 80 to 140 ms. Amplitudes were then averaged within a ±25 ms range around the peak, resulting in one N1 amplitude value per participant and task for the first click. The P2 component was identified similarly, with a peak search window of 150–250 ms and a ±25 ms range around the peak. For the second click, we added 500 ms to the N1 and P2 time‐window of the first click, and averaged across this time‐window. We performed a peak‐to‐peak analysis by subtracting the N1 amplitude from the P2 amplitude for each click. The resulting difference score defined the response amplitude for each click, sound condition, task, and participant. The difference between the first and second click defined the gating value.

### 2.6 TRF Analysis

#### 2.6.1 Audio Preprocessing

To relate the ongoing soundscape to the ongoing neural activity, we extracted the envelope of the OR playback and speech. Since the irrelevant speech was presented separately from the OR playback, we computed one TRF for the OR playback and one for the speech, instead of computing one TRF for the mixed signal. The OR playback included noise from running machines and ventilation, which produced an envelope with little variation. Such low variability in the envelope can lead to poor TRF estimation (Rosenkranz et al. 2023). To address this, we applied a Wiener filter implemented in MATLAB (Plapous et al. 2006; Scalart 2023). For this, we first high‐pass filtered each OR playback at 1 Hz (filter order = 1000, transition bandwidth = 0.5 Hz, cutoff frequency (−6 dB) = 0.00004 Hz). We then estimated the power spectral density of the noise using the first second of each OR playback, as it was representative of the static noise in the OR playback. The noise estimate was then subtracted from the remaining signal. Afterwards, we extracted the envelope from the noise‐reduced OR playbacks and raw speech using the mTRFenvelope function (Crosse et al. 2016, 2021). We resampled all envelopes to 125 Hz.

#### 2.6.2 Backward Modeling

A backward modeling approach was utilized to analyze the response to each stimulus envelope separately using the mTRF toolbox (Crosse et al. 2016, 2021). In backward (decoding) TRF modeling, a linear mapping from the multichannel EEG signal to a stimulus feature—in this case, the stimulus envelope—is estimated. The model learns a set of weights *w*_*c*_(*τ*) across EEG channels c and time lags τ, such that a weighted sum of time‐lagged EEG activity best reconstructs the stimulus envelope:

$$\hat{s}\left(t\right)=\sum_{c=1}^{c}\sum_{\tau =\tau_{\min}}^{\tau_{\max}}w\left(\tau \right)\;\;\mathrm{EE}G_{c}\left(t-\tau \right)$$

where $\hat{s}\left(t\right)$ denotes the reconstructed stimulus envelope. The decoder weights were estimated using ridge regression, with the regularization parameter selected via nested cross‐validation.

For compactness, the same model can be expressed in matrix form as follows:

$$\hat{s}=\mathrm{XW},$$

where X is the time‐lagged EEG design matrix of size T×F, with T denoting the number of time samples and F=C×L the number of channels–lag features. Each column of X corresponds to one EEG channel at a specific time lag, and W contains the decoder weights.

Model performance was quantified as the Pearson correlation between the reconstructed envelope $\hat{s}\left(t\right)$ and the true stimulus envelope $s\left(t\right)$ in the held‐out test data. This correlation coefficient reflects reconstruction (prediction) accuracy, rather than the amplitude of neural responses.

Following this, we implemented a nested cross‐validation procedure separately for each task and stimulus to estimate decoder performance while preventing overfitting. As a first step, the EEG data were resampled to 125 Hz to match the sampling rate of the envelopes. The first and last 5 s of the EEG data and envelope of each block was removed. Each block was split in half, resulting in six segments per task. The mean duration of each segment was 175 s for the peg transfer and 162 s for the suturing task. Each segment served once as a test set while the remaining segments served as training sets, resulting in six folds. For each fold, the following procedure was employed: The training segments were cross‐validated to determine the optimal regularization parameter (i.e., lambda) using the function *mTRFcrossval*. The optimal lambda was searched for in the range of 10e‐7 to 10e7. A time‐lag window from 0 to 300 ms was used in the backward TRF model, defining the range of stimulus–response latencies over which EEG activity was allowed to contribute to stimulus reconstruction. This lag range was chosen because neural responses to task‐irrelevant auditory stimuli typically occur at early latencies and reflect initial sensory and attentional processing (Hausfeld et al. 2018, 2021). The lambda corresponding to the maximum correlation coefficient was selected for model training. The model was then trained using the *mTRFtrain* function and the optimal lambda. The trained model was then applied to the test set using the *mTRFpredict* function, which correlated the actual and predicted stimulus envelope. This resulted in one Pearson correlation value (i.e., prediction accuracy) per fold which was averaged across folds, resulting in one correlation value per participant, task, and stimulus (see supplementary Figure S1 for an illustration of a reconstructed envelope). In all conditions, we analyzed the neural tracking of the OR playback. Additionally, in the speech‐present condition, we applied the same analysis procedure to the speech stimuli. For this, we computed TRFs using the combined speech material, disregarding differences in content, speaker sex, and speech characteristics, such as word frequency.

We also establish an empirical chance‐level reference for visualization purposes. For this, a permutation test was conducted using the *mTRFpermute* function. This involved shuffling the response data and recalculating the correlation to generate a distribution of correlation coefficients under the null hypothesis, representing chance‐level performance. In total, 17 permutations were performed per fold and averaged across folds, resulting in 102 permutations per participant, task, and stimulus. The permutation test of each fold used the optimal lambda of the respective fold. The chance level was defined as the 95th percentile across all permutations of a stimulus (Crosse et al. 2021).

### 2.7 Statistical Analyses

All statistical analyses were performed in R Studio (v4.2.1). For most outcome measures, we fitted a series of linear mixed models using the R packages lmer4 (v1.1‐30) (Bates et al. 2015). LMMs are particularly well‐suited for data with hierarchical structures, such as repeated measures within participants, as they allow for both fixed effects (effects of interest) and random effects (participant‐specific variability). We began by fitting a baseline model that included only the random intercept of the participant to account for individual differences in overall response levels. We subsequently added fixed then random effects and evaluated the improvement in model fit. The inclusion of random slopes was motivated by the possibility that the effect of the predictors might vary across participants. Allowing for this variation helps avoid biased estimates and improves generalizability. For most models, the predictors of *task* and/or *sound* were added. *Task* contained two categories, peg transfer and suturing, which were coded 0 and 1, respectively. *Sound* contained two categories, speech absent and speech present, which were coded 0 and 1, respectively. The best fitting model was determined using likelihood‐ratio testing. We report results from the likelihood‐ratio test comparing a model with a fixed or random effect to a model without the effect. We further report for the fixed effects the *b* value and standard error (SE) of the best fitting model. The model comparisons for all computed models can be found in Supporting Information (Section Model Comparisons).

#### 2.7.1 Self‐Reported Workload

For self‐reported workload, we used the total SURG‐TLX score, which was calculated by averaging the scores for all items. For each task and sound condition, the mean score was calculated across blocks. This resulted in 2 (task: peg transfer vs. suturing) × 2 (sound: speech present vs. speech absent) “total workload” scores for each participant. We expected the “total workload” score to change between the tasks and explored the effect of the sound condition. Therefore, we iteratively added the first *task*, second *sound*, and then their interaction as fixed effects. After fitting the fixed effects, we fitted the random effects by adding *task* and *sound* as random slopes.

Although the “total workload” score was our main outcome measure, we also explored how task and sound affect the different aspects of surgical demand. Therefore, we followed the same procedure as for the total score also for each item of the SURG‐TLX (i.e., mental demand, physical demand, temporal demand, complexity, stress, and distraction).

#### 2.7.2 Surgical Task Performance

We computed separate linear mixed models for the outcome measures of the peg transfer and suturing tasks. As the outcome parameters were different for each task, we did not investigate whether performance differed across tasks. Instead, we focused on whether the presence of speech influenced task performance. For each outcome parameter, we investigated the effect of adding the fixed and random slope of *sound*. The outcome measure “transfers” and “drops” were count variables, thus we investigated these using generalized linear mixed models with a Poisson distribution. For the outcome measure “duration” the data were skewed, as most participants did not complete the task within the 6 min. Therefore, we computed a beta mixed model (Verkuilen and Smithson 2012) using the R package glmmTMB (v.1.1.7). For this, we normalized all values between 0 and 1 with 1 indicating that the entire duration was used. As beta models do not allow values to be exactly 1 we transformed boundary values by subtracting 0.002. Otherwise, the same method as with the other outcome parameters was applied.

#### 2.7.3 Sensory Gating

We use the paired‐click paradigm to assess the amount of sensory gating for each task. We first evaluated whether a gating effect was present. We did this separately for the sound conditions, as they were acoustically different; thus, different responses could be expected. For each sound condition, we evaluated whether the response to the first and second click were different; in other words, whether gating was present. We computed a linear mixed model for each sound condition using the averaged response amplitudes to the clicks as an outcome measure. We chose to analyze averaged ERP data in order to maximize signal‐to‐noise ratio and to obtain stable estimates of component amplitudes, which is particularly important when dealing with noisy EEG data in mobile experiments. The baseline model included participants as a random intercept, and *position* (coded 0 and 1 for the response to the first and second click, respectively) was subsequently added as a fixed effect and random slope.

We then checked whether gating differed between tasks. The gating value was obtained by subtracting the amplitude of the first click from that of the second click. This gating value, calculated for each participant and task, reflects the strength of the sensory gating, with a larger value indicating a larger gating; in other words, better suppression of the second click after hearing the first one. For each sound condition, we computed the baseline model including participants as a random intercept and subsequently added *task* as a fixed effect and random slope.

#### 2.7.4 Continuous Stimuli

For the response to the continuous stimuli, we were interested in how the individual parts of the continuous stimulus were processed. Thus, we computed three prediction accuracies. For the speech‐absent condition, only the playback stimulus was modeled, resulting in one prediction accuracy. For the speech‐present condition, the playback and speech were modeled individually, resulting in two prediction accuracies. Each prediction accuracy was modeled using the baseline model participant as a random intercept and subsequently adding *task* as a fixed effect and random slope.

#### 2.7.5 Exploratory Analyses

We investigated the relationship between self‐report and neural measurements. To do this, we used the prediction accuracies (i.e., *r*) as continuous predictors for the “total workload” score. We calculated the average workload score for both the blocks where speech was absent or present. In both the speech‐absent and speech‐present conditions, the prediction accuracy of the OR playback was used to predict the “total workload” for each respective condition. In addition, in the speech‐present condition, the prediction accuracy of the speech stimulus was also used to predict the “total workload” for that condition. To receive meaningful *b* estimates we standardized the prediction accuracies for each stimulus. To account for the effect of task, *task* was also included as a predictor. Thus, the baseline model included the random effect participant and fixed effect *task*. We subsequently added the fixed effect *r* and the interaction between *r* and *task*. This was done for each stimulus separately.

#### 2.7.6 Multiple Comparison Correction

Evidence for an effect was assumed at *α* = 0.05. Because multiple models were often used to test the same hypothesis, we applied corrections for multiple comparisons based on the number of contrasts within each analysis. For self‐reports, we examined only one outcome measures (i.e., workload), thus no correction was applied. For surgical task performance, four outcome measures were analyzed, yielding *α* = 0.05/4. For ERPs, separate models were computed for speech‐absent and speech‐present conditions, leading to *α* = 0.05/2. Finally, for TRFs, models were computed for three stimulus types, resulting in *α* = 0.05/3.

## 3 Results

### 3.1 Self‐Reported Workload

#### 3.1.1 Total Score

**FIGURE 3:**
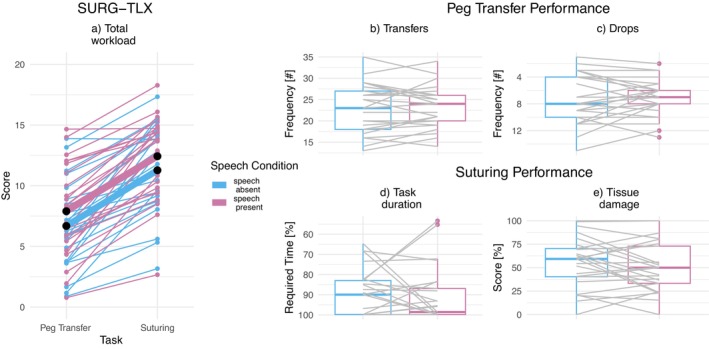
Observed scores for the self‐report and performance measures for each task and sound condition. The tasks had two difficulty levels, the easy peg transfer task and the difficult suturing task. Self‐reports were derived from the SURG‐TLX. (a) The “total workload” score (task & sound: *p* < 0.001). (b) the number of transferred rings, and (c) the number of dropped rings. For the suturing task we used (d) the amount of the total time that was required (i.e., 0–6 min, *M* = 5.4 min), and (e) the efficacy of handling the tissue. For all performance plots, a high *y*‐axis value indicates good performance, and a low value bad performance. The gray lines show individual participants. None of the performance measures was significantly affected by the sound condition.

Most participants reported a higher overall workload during the suturing compared to peg transfer task (Figure 3a). The best fitting model included the main effects *task* (*χ* ^2^(1) = 85.86, *p* < 0.001, *b* = 4.58, SE = 0.5) and *sound* (*χ* ^2^(1) = 12.01, *p* < 0.001, *b* = 1.2, SE = 0.27), but no interaction (*χ* ^2^(1) = 0.0001, *p* = 0.993). When allowing the effect of *task* and *sound* to vary across participants, the model fit further improved (task: *χ* ^2^(1) = 24.68, *p* < 0.001; sound: *χ* ^2^(1) = 10.26, *p* = 0.016). This indicates that participants experienced higher self‐reported workload during the suturing task compared to the peg transfer task, a higher workload when speech was present compared to when speech was absent, and that the strength of both effects varied between participants.

#### 3.1.2 Exploratory Analyses of Individual SURG‐TLX Items

We investigated the remaining SURG‐TLX items individually (see Figure S2) and listed the results in Table S1. To summarize, we subsequently added the fixed effects, *task*, *sound*, and their interaction, and the random slopes. Adding *task* as a fixed effect as well as a random slope significantly improved model fit for the items “mental demands,” “physical demands,” “temporal demands,” and “complexity of procedure.” For the same items, adding *sound* or the interaction between *task* and *sound* did not improve the model fit significantly. For the item “situational stress,” the best fitting model included the main effects for *task* and *sound* and random slopes for both effects. For the item “environmental distraction,” the best fitting model included only the main effects for *sound* and the random slope for *sound*. To summarize, whereas all items, except “environmental distraction,” showed higher scores for the suturing compared to peg transfer task, only the items “environmental distraction” and “stress” showed higher scores for the speech‐present compared to speech‐absent condition.

### 3.2 Surgical Task Performance

For the peg transfer task, the outcome parameters were the number of transfers and the number of drops. For the suturing task, the outcome parameters were the time required to complete the task and the extent of damage. As shown in Figure 3b–e, adding *sound* as a predictor did not change model performance for any outcome measure (Transfers: *χ* ^2^(1) = 0.008, *p* = 0.929; Drops: *χ* ^2^(1) = 0.25, *p* = 0.873; Time: *χ* ^2^(1) = 0.22, *p* = 0.64; Damage: *χ* ^2^(1) = 2.267, *p* = 0.132). This indicates that the presence of speech did not change the measured task performance during either surgical task. Furthermore, there was a high variability in performance between participants. This was probably the result of our participant pool that had little laparoscopic experience.

### 3.3 Sensory Gating

**FIGURE 4:**
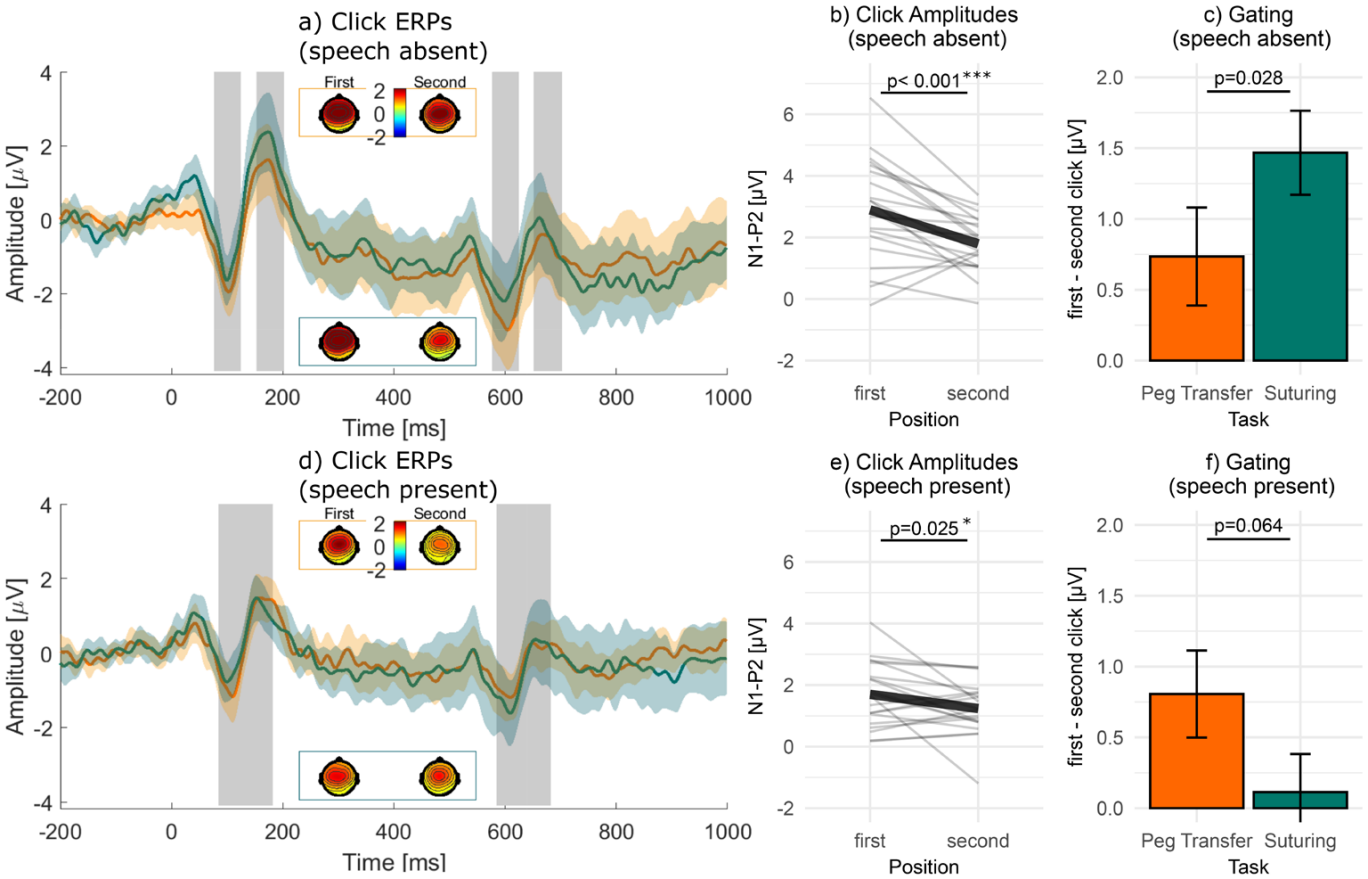
ERPs in response to the paired‐click paradigm. The top and bottom row show data for the speech‐absent and speech‐present condition, respectively. The first column (a, d) shows the ERP time‐course and topographies in response to the clicks, which were presented at 0 and 500 ms. The time‐course shows the averaged data across participants and channels FC1, FC2, Fz, and Cz (solid line), along with the confidence interval (shaded area) for each task. The gray areas highlight the N1 and P2 time windows. The topographies show the peak‐to‐peak difference from the averaged amplitudes in the N1 and P2 time‐window for the first and second click. The second column (b, e) shows the averaged amplitude for the first and second click. The thick line shows the observed average across participants, and the thin lines individual participants. The third column (c, f) shows the observed strength of gating, that is the difference between the response to the first and second click for each task (±1SE).

To investigate the presence of sensory gating, we computed whether the ERP amplitude changed from the first to the second click. We observed a gating effect in the speech‐present and speech‐absent condition. For both sound conditions, the model fit improved significantly when adding *position* as a predictor (Figure 4b,e; speech‐absent condition: *χ* ^2^(1) = 19.65, *p* < 0.001, *b* = −1.1, SE = 0.23; speech‐present condition: *χ* ^2^(1) = 5.03, *p* = 0.024, *b* = −0.46, SE = 0.2). Figure 4a,d show that the ERP amplitudes between the sound conditions are different, likely due to the different acoustics in each sound condition (i.e., the presence or absence of speech).

We then investigated whether the sensory gating strength changed between tasks. In the speech‐absent condition, adding *task* as a predictor did not significantly improve model fit after correction for multiple comparisons (Figure 4c; *χ* ^2^(1) = 4.836, *p* = 0.027, corrected *α* = 0.025, *b* = 0.732, SE = 0.32). Nevertheless, the estimated effect indicated a numerically larger N1–P2 peak‐to‐peak amplitude during the suturing task compared to the peg transfer task. Adding *task* as random slope led to an unidentifiable model. For the speech‐present condition, adding *task* as a predictor did not improve model fit, compared to the baseline model (Figure 4f; *χ* ^2^(1) = 344, *p* = 0.064). To summarize, we found a trend of stronger sensory gating in the suturing compared to peg transfer task in the speech‐absent condition.

### 3.4 Responses to Continuous Stimuli

**FIGURE 5:**
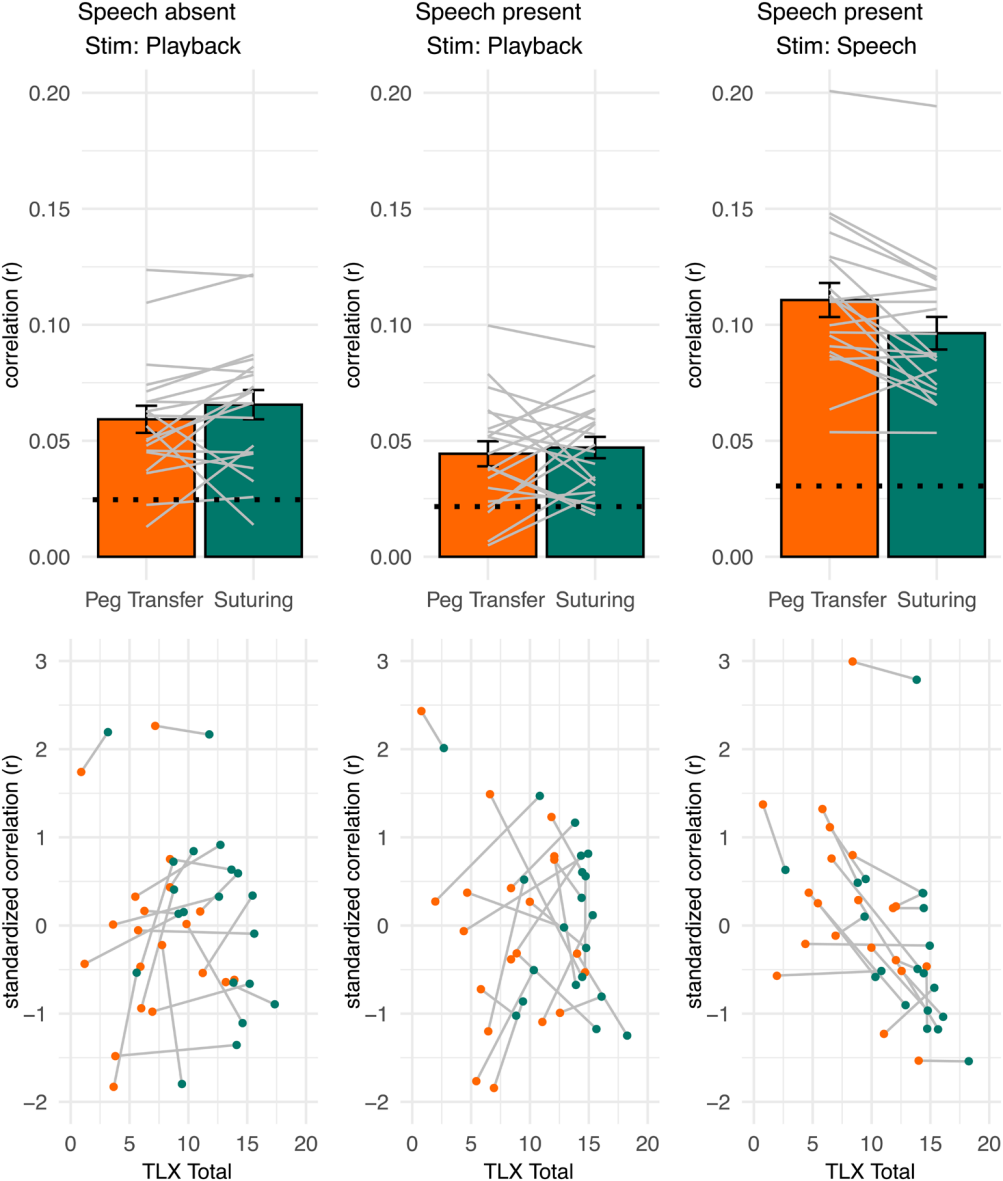
The plots show the effect of task on the correlation between the actual and reconstructed stimulus (top row) and the effect of task and standardized correlation coefficients on the “total workload” score (bottom row). Each column represents observed reconstruction accuracies for one of the three stimuli (i.e., Stim). Gray lines show data for individual participants. For the top row, the chance level (95th percentile of the permuted data) is represented by the black dotted line. For the bottom row, we predicted the workload score using the standardized correlation coefficients, but plotted the workload score on the *x*‐axis and centered correlation on the *y*‐axis, to visually match the top row.

Figure 5 (top row) shows that the average prediction accuracy of each model was above the 95th percentile derived from the permuted data, indicating that the stimulus was reliably encoded in the EEG signal (Crosse et al. 2021). For the OR playback, adding *task* as a predictor neither increased model performance when speech was absent (*χ* ^2^(1) = 2.08, *p* = 0.149) nor when speech was present (*χ* ^2^(1) = 0.27, *p* = 0.6). For the speech stimulus, adding *task* as a predictor improved model performance (*χ* ^2^(1) = 10.3, *p* = 0.001, *b* = −0.014, SE = 0.004), but adding *task* as random slope led to an unidentifiable model. This indicates that the prediction accuracy for speech was higher for the peg transfer than for the suturing task.

### 3.5 Relationship Between Self‐Report and Neural Tracking

We explored the relationship between the self‐report workload measures and neural tracking of the continuous stimuli (Figure 5, bottom row). Adding *r* as a predictor did not change model performance for the OR playback when speech was absent (*χ* ^2^(1) = 0.913, *p* = 0.334) nor when speech was present (*χ* ^2^(1) = 0.69, *p* = 0.406). However, adding *r* as a predictor significantly improved model performance for speech (*χ* ^2^(1) = 4.849, *p* = 0.027, *b* = −1.399, SE = 0.63), indicating that a lower neural tracking of speech, as reflected by reduced stimulus reconstruction accuracy, was associated with higher perceived workload. Adding the interaction of *task* and *r* did not improve the model further (*χ* ^2^(1) = 0.032, *p* = 0.857), and adding task or *r* as a random factor led to unidentifiable models.

## 4 Discussion

Although speech distraction in the OR is an often‐reported problem, only a few studies investigated this experimentally. Therefore, we studied how a soundscape, consisting of an OR playback, paired clicks, and irrelevant speech, is perceived and processed during the performance of an easy and difficult surgical task, namely peg transfer and suturing, respectively. To understand how the soundscape is processed and influences the individual, we employed self‐report, performance, and neurophysiological measurements.

Our findings highlight the distinction between objective and subjective distraction: while irrelevant speech did not impair surgical task performance (objective distraction), it reliably increased perceived workload and distraction ratings (subjective distraction). As expected, participants reported a higher workload for the (difficult) suturing task compared to the (easy) peg transfer task, consistent with previous research (Lim et al. 2023; Scerbo et al. 2017). In addition, the presence of speech further increased the perceived workload. The exploratory analysis of the individual SURG‐TLX items revealed that only the items' distraction and stress were influenced by the presence of speech. This pattern is in line with Weigl et al. (2015), who also found that both “Distraction” and “Stress” ratings increase in the presence of irrelevant speech. It is therefore plausible that irrelevant speech drove the increase in workload in our study. However, we cannot rule out that the effect was due to the mere presence of an additional sound source from behind the participant, such that any additional auditory input might have produced a similar increase in self‐reported distraction.

Although the exploratory analysis of participants' self‐reports indicates more distraction when speech was present, their surgical task performance remained unaffected by the presence of speech. Similarly, noise reduction interventions in the OR may increase subjective well‐being, without necessarily impacting patient outcomes (Engelmann et al. 2014; Leitsmann et al. 2021). We propose two potential explanations for this discrepancy. First, the maintenance of performance in the presence of task‐irrelevant speech comes at the cost of increased workload. This compensatory workload may have long‐term implications for surgeons' well‐being, even if patients are not immediately affected (Ayas et al. 2022). Second, performance measurements may not be sensitive enough to detect subtle differences in behavior. For instance, expert and novice surgeons can achieve a high performance on simple surgical tasks, even when distracted (Hsu et al. 2008). Furthermore, the evidence regarding the effects of noise on surgical performance is rather mixed (Mentis et al. 2016), which may be caused by the heterogeneity in performance measures across studies. This highlights the limitations of performance metrics which may only decline under severely distracting conditions, for instance, when several distractors are combined (Pluyter et al. 2010; Szafranski et al. 2009). Overall, our results suggest that while the measured performance may not suffer, irrelevant speech adds subjective mental strain. This suggests that improving the acoustic environment can benefit surgeons by reducing perceived workload, contributing to their overall well‐being.

We also investigated EEG as a third measure, complementing self‐reports and task performance. Unlike these behavioral measures, EEG provides access to covert neural processes that may reveal more subtle effects of the soundscape on the individual. To this end, we examined two well‐established measures from auditory neuropsychology and adapted them to our OR scenario: event‐related potentials (ERPs) to assess responses to transient paired clicks, and temporal response functions (TRFs) to assess responses to speech and continuous background sounds. These two approaches provide complementary insights into sound processing, and we explored to what extent they can be applied to understand sound perception in realistic scenarios.

Using ERPs, we investigated the early processing of the transient paired clicks and observed a reduced neural response to the second click compared to the first across all conditions. This can be taken as evidence that some form of sensory gating occurred. In the speech‐absent condition, gating strength tended to be larger during the more difficult task (suturing). Although this effect did not survive multiple comparison correction, the trend may suggest enhanced suppression of irrelevant auditory stimuli under high task demands. This would align with research showing that increased task demands strengthen early‐stage filtering mechanisms, thereby protecting cognitive functioning from irrelevant input (Bidet‐Caulet et al. 2015; Miller et al. 2011; Sörqvist et al. 2016, 2012; Lijffjt et al. 2009).

In the speech‐present condition, we found no significant effect of task demand on gating strength. We assume that the speech may have obscured the clicks, lowering their perceptibility and reducing ERP amplitudes (Brungart et al. 2001; Shinn‐Cunningham 2008). Indeed, in an earlier study Hölle and Bleichner (2023) also found that the presence of an additional auditory stimulus (speech) reduced ERP amplitudes in response to the paired‐click paradigm. As a consequence, the overall reduction in amplitude may have rendered the difference between the first and second click too small to reliably detect, particularly against the background noise inherent in mobile EEG recordings during a complex task. Alternatively, the speech may have engaged more perceptual resources than the clicks. Stimuli that are perceived as separate auditory objects, characterized by variations in spatial location or spectro‐temporal content (Griffths and Warren 2004), are also processed separately (Hausfeld et al. 2018). The separation may lead to a preference to process speech, thereby allocating fewer resources to the processing of the clicks (Shinn‐Cunningham 2008). This in turn limited the gating effect. Future research could explore these explanations by investigating how irrelevant sounds influence the processing of each other.

For the OR playback, we found that the soundscape could be decoded from the neural data, but contrary to our hypothesis, its processing was not modulated by task demand. One possible explanation is that our analytic definition of the OR playback as a single auditory object did not reflect participants' actual perception. Although clicks and speech can reasonably be treated as distinct auditory objects with consistent acoustic properties (Shinn‐Cunningham 2008), the OR playback likely consisted of many different objects, each of which may have elicited different neural responses and task modulations (Huang and Elhilali 2020). By treating the playback as a unitary object, we may have overlooked such effects. Another explanation is that our analysis approach may have biased the results toward speech: to ensure comparability, we used the temporal envelope as a feature for both speech and OR playback. Although the envelope is suitable for continuous sounds (Di Liberto et al. 2020; Hausfeld et al. 2018; Rosenkranz et al. 2023, 2024), it is especially effective for maximizing speech responses (Crosse et al. 2016), whereas alternative representations, such as mel‐spectrograms, capture more variance in neural data (Di Liberto et al. 2015; Haupt et al. 2025). Such approaches may therefore be more sensitive to detect task‐related modulations. More generally, when dealing with complex soundscapes containing multiple overlapping sounds, analyses that differentiate sounds by content or acoustic features may provide a more detailed picture of how they are processed.

For the speech stimulus, we found a reduction in speech processing under high task demand. Interestingly, the direction of this effect is similar to the observed (but not significant) effect for the paired clicks in the speech‐absent condition. One possible interpretation is that the most prominent auditory stream is subject to modulation by task demand: when no speech is present, the clicks constitute the dominant stimulus and show gating effects. In the presence of speech, it is speech that becomes the dominant stream and shows reduced processing. This suggests that the auditory system flexibly allocates resources to the most salient stimulus in the soundscape, and that task demand primarily impacts processing of this most salient auditory object.

The speech suppression effect observed in the TRF analyses reflects modulation of neural processing within an early stimulus–response latency range, as the backward model was constrained to time lags between 0 and 300 ms. This lag range captures neural activity that contributes to the encoding and attentional modulation of continuous auditory input (e.g., Hausfeld et al. 2018). The present findings are therefore consistent with speech‐processing studies using dual‐ and multi‐talker paradigms, which suggest that ignored speech is attenuated at early stages of auditory processing (Hausfeld et al. 2018, 2021). Together, these results indicate that filtering of irrelevant speech in the present task likely occurs during early auditory and attentional processing stages. In addition, our exploratory analyses revealed an inverse relationship between neural responses to irrelevant speech and self‐reported workload, even when accounting for task difficulty. In other words, participants who showed stronger neural tracking of irrelevant speech tended to report lower workload, suggesting that they may have experienced the tasks as less demanding overall and therefore had attentional resources available to allocate to irrelevant speech.

Although neurophysiological measures are increasingly used to assess workload, they often rely on ERPs elicited by artificial and repetitive sounds, which are rarely encountered in real‐world environments (Wascher et al. 2021). We demonstrated that neural tracking of naturalistic stimuli, such as speech, can also serve as an indicator of perceived workload. This finding could encourage future studies investigating complex work environments to use natural stimuli.

Whilst the present study systematically examined the processing of irrelevant speech and its effect on task performance, certain limitations should be noted. The short duration of the tasks, the performance metrics used, and the relatively high variability of performance in the sample of inexperienced participants may have limited the ability to detect subtle performance impacts (Mentis et al. 2016). Furthermore, the ability to react to the soundscape is likely to change with experience (Hsu et al. 2008). As the present study focused on participants with no prior experience of the OR, the results may reflect more general demand modulations on auditory processing that are susceptible to change with experience. Hence, future studies utilizing longer tasks and experienced personnel could offer further insights into the impact of speech distraction on surgical personnel. We also acknowledge a limitation related to our use of linear mixed‐effects models on aggregated ERP data. Although averaging improves signal‐to‐noise ratio and yields robust component estimates, it reduces the number of observations available for model fitting. As a consequence, several more complex models, particularly those including random slopes and interactions, failed to converge. This likely reflects insufficient data to reliably estimate participant‐specific slopes. Regarding the TRF approach, it should be noted that the reconstructed envelope from the backward linear decoder is an unconstrained linear prediction and is therefore not expected to reproduce the bounded, right‐skewed distribution of the acoustic envelope (as can be seen in supplementary Figure S1). This should be considered when interpreting absolute reconstruction correlations, but does not by itself indicate biased model estimation.

In the effort to understand auditory distraction in a real‐world setting, we combined self‐reports, performance, and neurophysiological measures. We investigated how a complex soundscape, consisting of an OR playback, paired clicks, and irrelevant speech, is perceived and processed during the performance of surgical tasks of varying difficulty. Our study demonstrates that while irrelevant speech may not immediately impact performance, it increases perceived workload during difficult tasks. This finding may generalize to other high‐stakes settings where controlling the auditory environment is necessary to support the well‐being of personnel.

Our results indicate that irrelevant speech increased subjective distraction and workload during difficult tasks, even though objective distraction, reflected in surgical task performance, was unchanged. This highlights the complexity of finding subjective demand effects in performance for naturalistic tasks. Importantly, our results show that task demand changes the processing of sounds: transient clicks showed a trend toward stronger gating, and responses to irrelevant speech were reduced, suggesting enhanced filtering of irrelevant auditory input when cognitive demands are high. Furthermore, our study adds to the growing body of research that applies EEG beyond the lab and at workplaces (e.g., Dehais et al. 2020; Grasso‐Cladera et al. 2024; Wascher et al. 2021). Using mobile EEG, we could investigate the neurophysiological mechanisms underlying the processing of irrelevant aspects of the soundscape without interfering with task performance. We showed that irrelevant speech responses were reduced at an early stage of processing during demanding tasks and that this reduction was inversely related to self‐reported workload. Neurophysiological measures are often based on artificial and repetitive sounds (Wascher et al. 2021), but our results demonstrate that responses to naturalistic speech can also serve as sensitive markers of perceived workload. This highlights the potential of using mobile EEG with naturalistic stimuli to assess workload in complex, real‐world environments, paving the way for strategies to monitor and mitigate auditory distraction in high‐stakes contexts such as the OR.

## Author Contributions

**Verena N. Uslar:** conceptualization, resources, writing – review and editing. **Dirk Weyhe:** conceptualization, funding acquisition, resources, writing – review and editing. **Martin G. Bleichner:** conceptualization, funding acquisition, resources, supervision, writing – review and editing. **Marc Rosenkranz:** conceptualization, data curation, investigation, formal analysis, writing – review and editing, writing – original draft.

## Funding

This work was funded by the Deutsche Forschungsgemeinschaft (DFG, German Research Foundation) ID 411333557 and 490839860, and by the Forschungspool Funding of the Oldenburg School of Medicine and Health Science.

## Ethics Statement

This study involving human participants was reviewed and approved by Medizinische Ethikkommission, Carl von Ossietzky Universität Oldenburg, Oldenburg (2021‐031).

## Conflicts of Interest

The authors declare no conflicts of interest.

## Supporting information


**Figure S1:** The figure illustrates the TRF data of one participant from a single held‐out test segment in one cross‐validation fold. The upper panel presents three envelopes of a 180 s segment: a stimulus envelope of the playback (blue), its reconstruction from the EEG signal (orange), and an envelope from another segment of the playback (green), normalized and vertically offset (+6 for the true envelope and −6 for the non‐matching envelope) for visualization. Right panels show density‐coded scatter plots comparing the reconstructed envelope with the matching envelope (top) and non‐matching envelope (bottom), with Pearson correlations indicated in each panel. The three lower panels display 2‐s excerpts from the same segment to illustrate the temporal correspondence between the three signals in greater detail.
**Figure S2:** Score for each item of the SURG‐TLX for each task and sound condition. The tasks were selected to represent two difficulty levels, with the peg transfer task representing the easy task and suturing representing the difficult task. Score for each item of the SURG TLX for each task and sound condition. (a–d) A significant effect of task, nut not effect of sound condition or an interaction effect. (e, f) A significant effect of task and sound condition, but no interaction effect. The thin lines show the participants' data for each sound condition, the thick line the average across participants.
**Table S1:** SURG‐TLX total score and items.
**Table S2:** Surgical task performance.
**Table S3:** ERP: Presence of gating.
**Table S4:** ERP: Gating difference between tasks.
**Table S5:** TRF: Difference between tasks.
**Table S6:** TRF: prediction of workload.
**Table S7:** Descriptive data summary.

## Data Availability

The data that support the findings of this study are openly available in Zenodo at http://doi.org/10.5281/zenodo.14899817.
