# Supplementary material for: The Effect of Task Demand on EEG Responses to Irrelevant Sound and Speech in Simulated Surgical Environments
Source: Psychophysiology. 2026 Jul 14;63(7):e70360. doi: 10.1111/psyp.70360 (PMC13370042; doi:10.1111/psyp.70360)
Supplement: Supplementary file 1 — Figure S1: The figure illustrates the TRF data of one participant from a single held‐out test segment in one cross‐validation fold. The upper panel presents three envelopes of a 180 s segment: a stimulus envelope of the playback (blue), its reconstruction from the EEG signal (orange), and an envelope from another segment of the playback (green), normalized and vertically offset (+6 for the true envelope and −6 for the non‐matching envelope) for visualization. Right panels show density‐coded scatter plots comparing the reconstructed envelope with the matching envelope (top) and non‐matching envelope (bottom), with Pearson correlations indicated in each panel. The three lower panels display 2‐s excerpts from the same segment to illustrate the temporal correspondence between the three signals in greater detail. Figure S2: Score for each item of the SURG‐TLX for each task and sound condition. The tasks were selected to represent two difficulty levels, with the peg transfer task representing the easy task and suturing representing the difficult task. Score for each item of the SURG TLX for each task and sound condition. (a–d) A significant effect of task, nut not effect of sound condition or an interaction effect. (e, f) A significant effect of task and sound condition, but no interaction effect. The thin lines show the participants' data for each sound condition, the thick line the average across participants. Table S1: SURG‐TLX total score and items. Table S2: Surgical task performance. Table S3: ERP: Presence of gating. Table S4: ERP: Gating difference between tasks. Table S5: TRF: Difference between tasks. Table S6: TRF: prediction of workload. Table S7: Descriptive data summary. [file PSYP-63-e70360-s001.pdf]

Supplementary Material

TRF illustration

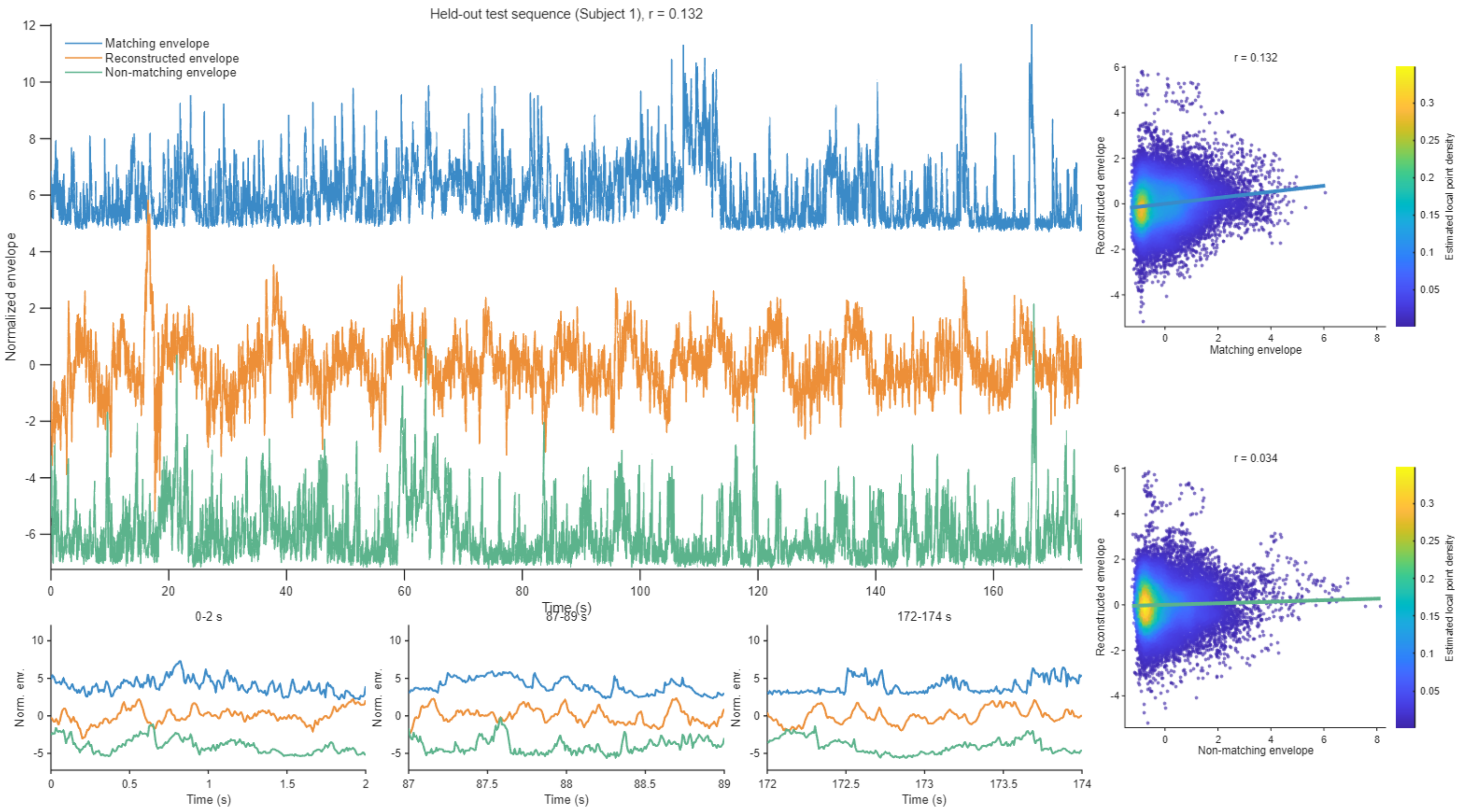

Figure 1: The figure illustrates the TRF data of one participant from a single held-out test segment in one cross-validation fold. The upper panel presents three envelopes of a 180 s segment: a stimulus envelope of the playback (blue), its reconstruction from the EEG signal (orange), and an envelope from another segment of the playback (green), normalized and vertically offset (+6 for the true envelope and −6 for the non-matching envelope) for visualization. Right panels show density-coded scatter plots comparing the reconstructed envelope with the matching envelope (top) and non-matching envelope (bottom), with Pearson correlations indicated in each panel. The three lower panels display 2-s excerpts from the same segment to illustrate the temporal correspondence between the three signals in greater detail.

SURG-TLX items

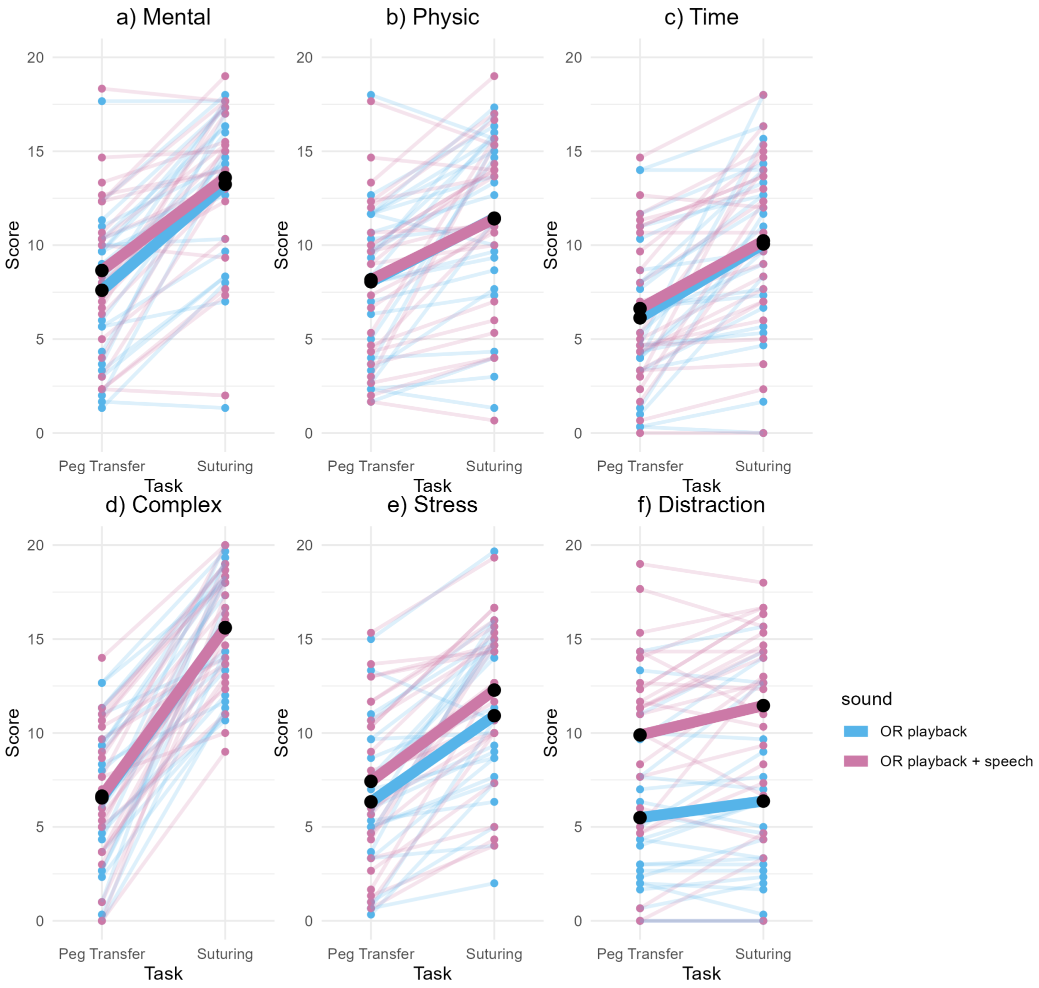

Figure 2: Score for each item of the SURG-TLX for each task and sound condition. The tasks were selected to represent two difficulty levels, with the peg transfer task representing the easy task and suturing representing the difficult task. Score for each item of the SURG-TLX for each task and sound condition. a)-d) showed a significant effect of task, nut not effect of sound condition or an interaction effect. e)-f) showed a significant effect of task and sound condition, but no interaction effect. The thin lines show the participants' data for each sound condition, the thick line the average across participants.

Model comparisons

Table 1: *SURG-TLX total score and items*

Model Descriptions:

M0: $\hat{y} \sim 1 + (1|participant)$
M1: $\hat{y} \sim task + (1|participant)$
M2: $\hat{y} \sim task + sound(1|participant)$
M3: $\hat{y} \sim task * sound + (1|participant)$
M4: $\hat{y} \sim task + (task|participant)$
M5: $\hat{y} \sim task + sound + (task|participant)$
M6: $\hat{y} \sim task + sound + (task + sound|participant)$
M7: $\hat{y} \sim sound + (1|participant)$
M8: $\hat{y} \sim sound + (sound|participant)$

**Outcome**	**Model Comparison**	**AIC**	**Chisq**	**p-value**	**Fixed effects (Estimate ± SE)**	$\mathbf{R}^2$
	M0	556.87				
	M1 vs M0	473.01	85.86	$< 2.2e-16$ ***	Intercept: 6.69 ± 0.70	0.308
Total	M2 vs M1	462.99	12.01	0.0005 ***	Task (SU): 4.58 ± 0.50	
	M3 vs M2	464.99	0.0001	0.9934	Sound (Sp): 1.20 ± 0.27	
	M5 vs M2	442.31	24.68	4.37e-06 ***		
	**M6** vs M5	438.05	10.26	0.016 *		
	M0	558.36				
Mental	M1 vs M0	515.32	75.04	$< 2.2e-16$ ***	Intercept: 8.13 ± 0.77	0.301
	M2 vs M1	514.92	2.39	0.1219	Task (SU): 5.30 ± 0.66	
	**M4** vs M1	504.79	14.52	0.0007 ***		
	M0	548.69				
Physical	M1 vs M0	503.28	47.41	5.75e-12 ***	Intercept: 8.11 ± 0.82	0.124
	M2 vs M1	505.26	0.02	0.8965	Task (SU): 3.33 ± 0.64	
	**M4** vs M1	471.30	35.98	1.54e-08 ***		
	M0	559.65.69				
Time	M1 vs M0	508.78	52.86	3.58e-13 ***	Intercept: 6.38 ± 0.77	0.166
	M2 vs M1	510.09	0.69	0.4054	Task (SU): 3.80 ± 0.68	
	**M4** vs M1	478.05	34.73	2.87e-08 ***		
	M0	636.35				
Complexity	M1 vs M0	495.29	143.07	$< 2.2e-16$ ***	Intercept: 6.59 ± 0.75	0.63
	M2 vs M1	497.27	0.02	0.9025	Task (SU): 9.03 ± 0.74	
	**M4** vs M1	419.71	79.58	$< 2.2e-16$ ***		
	M0	580.57				
	M1 vs M0	517.15	65.43	6.04e-16 ***	Intercept: 6.25 ± 0.79	0.259
Stress	M2 vs M1	511.49	7.66	0.0056 **	Task (SU): 4.75 ± 0.62	
	M3 vs M2	513.35	0.131	0.716	Sound (Sp): 1.25 ± 0.43	
	M5 vs M2	501.91	13.57	0.0011 **		
	**M6** vs M5	493.67	14.25	0.0026 **		
	M0	592.94				
Distraction	M1 vs M0	591.98	2.96	0.085	Intercept: 5.93 ± 0.87	0.201
	M7 vs M0	534.64	60.298	8.15e-15 ***	Sound (Sp): 4.79 ± 0.69	
	**M8** vs M7	525.44	13.204	0.00136 **		

*Model comparisons for the SURG-TLX total and individual scores. The distraction item can be found in the next table, as the model computation differed for this item from that of the other items. The fixed effects are estimated from the final model, marked in bold. The marginal R^2 represents the effect size of all predictots in the final model. Model descriptions provided at the top of the table. Note, that M4 was only tested, if M2 was not significant, and M7 was only tested if M1 was not significant. SU: Suturing, Sp: Speech present. Significance levels: * $p < .05$, ** $p < .01$, *** $p < .001$.*

Table 2: *Surgical task performance*

Outcome	Model Comparison	AIC	Chisq	p-value	Fixed effects (Estimate ± SE)	R^2
**Model Descriptions:**						
M0: $\hat{y} \sim 1 + (1\|participant)$						
M1: $\hat{y} \sim sound + (1\|participant)$						
PT-Transfers	M0	301.48				
	M1 vs **M0**	303.47	0.008	0.929	Intercept: 3.125 ± 0.04	0
PT-Drops	M0	250.55				
	M1 vs **M0**	252.52	0.25	0.873	Intercept: 1.9 ± 0.08	0
SU-Duration	M0	-168.76				
	M1 vs **M0**	-166.98	0.2195	0.64	Intercept: 2.35 ± 0.26	0
SU-Damage	M0	456.76				
	M1 vs **M0**	456.49	2.2687	0.132	Intercept: 52.673 ± 5.016	0

*Model comparisons for the surgical task performance. Model descriptions provided at the top of the table. The fixed effects are estimated from the final model, marked in bold. The marginal R^2 represents the effect size of all predictots in the final model. PT: Peg transfer. SU: Suturing. Significance levels: * $p < .05$, ** $p < .01$, *** $p < .001$.*

Table 3: *ERP: Presence of gating*

Outcome	Model Comparison	AIC	Chisq	p-value	Fixed effects (Estimate ± SE)	R^2
**Model Descriptions:**						
M0: $\hat{y} \sim 1 + (1\|participant)$						
M1: $\hat{y} \sim position + (1\|participant)$						
M2: $\hat{y} \sim position + (click\|participant)$						
amp (Sa)	M0	289.62				
	**M1** vs M0	271.97	19.651	9.296e-06 ***	Intercept: 2.89 ± 0.294	0.119
	M2 vs M1	singularity	issue		position (2nd): -1.1 ± 0.23	
amp (Sp)	M0	244.35				
	**M1** vs M0	241.31	5.03	0.024 *	Intercept: 1.69 ± 0.211	0.04
	M2 vs M1	convergence	failure		position (2nd): -0.46 ± 0.2	

*Model comparisons to check whether a gating effect is present. The N1-P2 peak-to-peak amplitude was estimated seperately for the 'speech present' and 'speech absent' condition. Model descriptions provided at the top of the table. The fixed effects are estimated from the final model, marked in bold. The marginal R^2 represents the effect size of all predictots in the final model. Sa: Speech absent, Sp: Speech present. Significance levels: * $p < .05$, ** $p < .01$, *** $p < .001$.*

Table 4: *ERP: Gating difference between tasks*

Outcome	Model Comparison	AIC	Chisq	p-value	Fixed effects (Estimate ± SE)	R^2
**Model Descriptions:**						
M0: $\hat{y} \sim 1 + (1\|participant)$						
M1: $\hat{y} \sim task + (1\|participant)$						
M2: $\hat{y} \sim task + (task\|participant)$						
gating (Sa)	M0	145.71				
	**M1** vs M0	142.88	4.836	0.0279 *	Intercept: 0.735 ± 0.32	0.062
	M2 vs M1 (did not converge)				Task (SU): 0.732 ± 0.32	
gating (Sp)	M0	140.52				
	M1 vs **M0**	139.08	3.44	0.064	Intercept: 0.46 ± 0.222	0

*Model comparisons to investigate whether the strength of gating differed between tasks. Model descriptions provided at the top of the table. The fixed effects are estimated from the final model, marked in bold. The marginal R^2 represents the effect size of all predictots in the final model. PT: Peg transfer, SU: Suturing, Sa: Speech absent, Sp: Speech present. Significance levels: * $p < .05$, ** $p < .01$, *** $p < .001$.*

Table 5: *TRF: Difference between tasks*

Outcome	Model Comparison	AIC	Chisq	p-value	Fixed effects (Estimate ± SE)	R^2
**Model Descriptions:**						
M0: $\hat{y} \sim 1 + (1 \| participant)$						
M1: $\hat{y} \sim task + (1 \| participant)$						
M2: $\hat{y} \sim task + (task \| participant)$						
playback response (Sa)	M0	-184.83				
	M1 vs **M0**	-184.91	2.08	0.149	Intercept: 0.062 ± 0.006	0
playback response (Sp)	M0	-190.54				
	M1 vs **M0**	-188.81	0.27	0.6	Intercept: 0.046 ± 0.004	0
speech response (Sp)	M0	-172.38				
	**M1** vs M0	-180.38	10.3	0.001 **	Intercept: 0.11 ± 0.008	0.048
	M2 vs M1	convergence	failure		Task (SU): -0.014 ± 0.004	

*Model comparisons to investigate whether the tasks influenced the processing of the different continuous stimuli. Model descriptions provided at the top of the table. The fixed effects are estimated from the final model, marked in bold. The marginal R^2 represents the effect size of all predictots in the final model. SU: Suturing, Sa: Speech absent, Sp: Speech present. Significance levels: * $p < .05$, ** $p < .01$, *** $p < .001$.*

Table 6: ***TRF: prediction of workload***

Outcome	rs	Model Comparison	AIC	Chisq	p-value	Fixed effects (Estimate ± SE)	R^2
**Model Descriptions:**							
M0: $\hat{y} \sim task + (1 \| participant)$							
M1: $\hat{y} \sim task + rs + (1 \| participant)$							
M2: $\hat{y} \sim task * rs + (1 \| participant)$							
TLX total	Playback response (Sa)	M0	211.29			Intercept: 7.02 ± 0.8	0
		M1 vs **M0**	212.36	0.913	0.334	Task (SU): 4.8 ± 0.68	
TLX total	Playback response (Sp)	M0	208.94			Intercept: 8.35 ± 0.83	0
		M1 vs **M0**	210.25	0.69	0.406	Task (SU): 4.63 ± 0.61	
TLX total	speech response (Sp)	M0	208.94			Intercept: 8.67 ± 0.78	0.385
		M1 vs M0	206.09	4.849	0.027 *	Task (SU): 4.01 ± 0.66	
		M2 vs **M1**	208.06	0.0322	0.857	rc: -1.399 ± 0.632	

*Model comparisons to investigate whether the standardized correlation (rs) between the actual and reconstructed stimulus envelope predict self-reported workload. Model descriptions provided at the top of the table. The fixed effects are estimated from the final model, marked in bold. The marginal R^2 represents the effect size of all predictots in the final model. SU: Suturing, Sa: Speech absent, Sp: Speech present. Significance levels: * $p < .05$, ** $p < .01$, *** $p < .001$.*

Table 7: *Descriptive data summary*

**Outcome**		**Sound**	
		**Sa**	**Sp**
TLX-total	SU	11.3 (3.59)	12.5 (3.44)
	PT	6.69 (3.47)	7.9 (3.75)
TLX-Mental	SU	13.2 (4.2)	13.6 (3.87)
	PT	7.6 (3.92)	8.65 (4.19)
TLX-Physical	SU	11.4 (4.74)	11.4 (4.65)
	PT	8.05 (4.14)	8.16 (4.27)
TLX-Time	SU	10.1 (4.5)	10.3 (4.62)
	PT	6.13 (3.91)	6.63 (4.07)
TLX-Complexity	SU	15.6 (3.16)	15.6 (3.13)
	PT	6.55 (3.86)	6.64 (3.71)
TLX-Stress	SU	10.9 (4.43)	7.43 (4.36)
	PT	6.33 (3.86)	7.43 (4.36)
TLX-Distraction	SU	5.49 (4.06)	9.89 (5.18)
	PT	6.37 (4.83)	11.5 (4.86)
PT-Transfers	SU		
	PT	23 (5.74)	23.1 (4.86)
PT-Drops	SU		
	PT	7.16 (3.7)	7.04 (2.76)
SU-Duration	SU	89.8 (11.6)	90.9 (13.8)
	PT		
SU-Damage	SU	55.6 (27)	49.8 (26.6)
	PT		
ERP N1-P2 first click	SU	3.03 (1.68)	1.51 (1.12)
	PT	2.76 (1.92)	1.89 (1.25)
ERP N1-P2 second click	SU	1.57 (0.98)	1.39 (1.14)
	PT	2.02 (1.25)	1.09 (1.03)
ERP Gating	SU	1.47 (1.33)	0.114 (1.2)
	PT	0.735 (1.55)	0.806 (1.37)
TRF playback	SU	0.066 (0.282)	0.047 (0.021)
	PT	0.059 (0.026)	0.044 (0.024)
TRF speech	SU		0.096 (0.31)
	PT		0.111 (0.33)

Descriptive statistics of each value used in the analysis. For each outcome measure the mean and standard deviation (in brackets) is provided. An empty cell indicates that no value exists for a certain outcome measure. SU: Suturing, PT: Peg Transfer, Sp: Speech present, Sa: Speech absent
